# A multi‐dimensional context‐dependent measurement framework for affective experience in preclinical AD

**DOI:** 10.1002/alz.086272

**Published:** 2025-01-03

**Authors:** Adam Turnbull

**Affiliations:** ^1^ Stanford University, Stanford, CA USA

## Abstract

Promoting emotional well‐being (EWB) in older adults at risk for Alzheimer’s Disease (AD), for example those with mild behavioral impairment (MBI), mild cognitive impairment (MCI), or subjective cognitive decline (SCD), is important both to improve quality of life and slow the progress of cognitive decline. Understanding how the early accumulation of AD pathology affects EWB and developing interventions to improve EWB both require the precise measurement of affective experience that plays a key role in EWB. Day to day affective experiences, both positive and negative, contribute significantly to EWB, but how affective experience maps onto EWB is complex. Rather than merely reflecting a decrease in negative affect or an increase in positive affect, we propose that EWB is increased when individuals have a full affective experience, moving through the high‐dimensional space of human emotion in a context‐appropriate manner in response to internal and external triggers. Understanding affective experience through a multi‐dimensional context‐dependent lens is therefore critical to assessing changes caused by early AD pathology. For example, irritability in an individual with MCI may only emerge in specific contexts (e.g., when attempting to perform a mentally challenging task) and may be reflected as a subtle increase in arousal. To understand 1) how affective experience contributes to EWB in older adults at‐risk for AD and 2) how early AD pathology accumulation affects EWB via changes in affective experience, we therefore need measurement techniques that accurately reflect the context‐dependent, high‐dimensional nature of affective experience. We propose a multi‐dimensional context‐dependent measurement framework for quantifying affective experience in the preclinical stages of AD. We demonstrate how this framework uses experience sampling to measure multi‐dimensional affective experience across a range of experimentally induced (social scenario paradigm) and real‐world (ecological momentary assessment) contexts. We explain how this framework can be used to better characterize affective experience in MBI, MCI, and SCD with the goals of 1) better developing interventions to improve EWB and slow cognitive decline and 2) better understanding how early AD pathology affects EWB via affective experience.

